# Hormonal and Osmoregulatory Responses in Intraoperative High-Volume Diuresis During Off-Pump Coronary Artery Bypass Grafting: An Exploratory Cohort Study

**DOI:** 10.3390/jcm14238395

**Published:** 2025-11-26

**Authors:** Yuxi Hou, Shuwen Li, Fei Cai, Fangyi Luo, Jun Ma

**Affiliations:** Beijing Anzhen Hospital, Capital Medical University, Beijing 100029, China; houyuxi1013@sina.com (Y.H.); dotocrshuwen@163.com (S.L.); 13051809977@163.com (F.C.); fangyi1346@163.com (F.L.)

**Keywords:** intraoperative high-volume diuresis, polyuria, off-pump coronary artery bypass grafting (OPCABG), copeptin, antidiuretic hormone (ADH), aldosterone, N-terminal pro–B-type natriuretic peptide (NT-proBNP), osmolality, diabetes insipidus (DI)

## Abstract

**Background**: Intraoperative high-volume diuresis is a frequent but underrecognized complication in cardiac surgery, potentially leading to hypovolemia, electrolyte imbalances, and hemodynamic instability. Its mechanisms remain poorly defined. This study investigated the hormonal and biochemical regulation of urine output during off-pump coronary artery bypass grafting (OPCABG). **Methods**: For this single-center observational cohort study, 70 patients undergoing OPCABG were enrolled (diuresis: urine output > 5 mL/kg/h, n = 38; normal, n = 32). Hormonal markers and osmolality parameters were measured perioperatively. Logistic regression was used to identify independent predictors, and receiver operating characteristic (ROC) curves was used to assess model performance. **Results**: Intraoperative high-volume diuresis occurred in 54.2% of patients. Logistic regression identified a low Body Mass Index (BMI) (OR 0.72, *p* = 0.002), reduced albumin (OR 0.75, *p* = 0.014), and lower copeptin (OR 0.43, *p* = 0.037) as independent predictors (AUC 0.855). Perioperatively, NT-proBNPT0 rose in both groups, aldosterone increased only in the diuresis group, and copeptin showed a slight nonsignificant rise. Plasma sodium was higher in cases of diuresis at the end of surgery (148.4 vs. 144.9 mmol/L, *p* < 0.001). Despite greater urine output and fluid infusion, the rates of intensive care unit (ICU) admission and hospital stays were similar. **Conclusions**: Intraoperative high-volume diuresis in OPCABG is strongly associated with reduced antidiuretic hormone activity, suggesting a partial central diabetes insipidus-like mechanism. Although not affecting short-term outcomes, it posed challenges for intraoperative fluid and electrolyte management. Larger multicenter studies are needed for validation.

## 1. Introduction

Intraoperative high-volume diuresis is a relatively common complication in cardiothoracic surgery [1]; however, it has received insufficient attention in clinical practice. Its potential consequences include intravascular volume depletion, electrolyte disturbances, hypotension, and even circulatory failure, all of which may adversely affect perioperative outcomes. Due to the suppression of sympathetic reflexes and vasodilation under general anesthesia, patients exhibit a diminished capacity for autonomic compensation in response to rapid fluid loss. Consequently, in the presence of intraoperative high-volume diuresis, anesthesiologists are often required to administer substantial fluid replacement or vasoactive agents to maintain hemodynamic stability [2].

High-volume diuresis results from excessive urine output driven by one or a combination of the following: impaired antidiuretic hormone activity (water diuresis), increased renal solute excretion (osmotic diuresis), or volume expansion-induced natriuretic mechanisms. Research on excessive intraoperative urine output has mainly focused on drug-induced perioperative diabetes insipidus (DI) [3] and postoperative DI following pituitary tumor surgery [4,5]. Retrospective studies have also reported increased intraoperative urine output in thoracic surgery [1]; However, in the field of cardiac surgery, evidence remains limited to isolated case reports [6,7,8]. In our preliminary retrospective analysis of off-pump coronary artery bypass grafting (OPCABG) performed at Anzhen Hospital between January and June 2023, the incidence of intraoperative high-volume diuresis was found to be 33%. Building upon these findings, the present study aimed to investigate the changes in plasma and urine osmolality and the associated hormonal regulation during intraoperative high-volume diuresis in OPCABG patients, seeking to explore the underlying mechanisms of diuresis and provide a theoretical basis for future preventive and therapeutic strategies.

## 2. Materials and Methods

This was a single-center observational cohort study conducted at Beijing Anzhen Hospital, Capital Medical University, between June 2025 and August 2025. It was an exploratory study designed to investigate the relationship between perioperative hormonal changes and the occurrence of excessive intraoperative urine output in patients undergoing OPCABG.

This study was not defined as an intervention clinical trial requiring prospective trial registration. The protocol was reviewed and approved by the Ethics Committee of Beijing Anzhen Hospital (approval no. KS2025058). Written informed consent was obtained from all participants prior to enrollment. The study was conducted in accordance with the Declaration of Helsinki and reported following the STROBE (Strengthening the Reporting of Observational Studies in Epidemiology) guidelines. The data set supporting the findings of this study is available from the corresponding author upon reasonable request.

### 2.1. Participants

Patients aged 18–75 y and undergoing off-pump coronary artery bypass grafting (OPCABG) were consecutively included. The exclusion criteria covered patients and control subjects with a pre-existing moderate to severe renal impairment (estimated glomerular filtration rate (eGFR) < 60 mL/min/1.73 m^2^), endocrine disorders affecting fluid balance, preoperative oral diuretics, perioperative use of intra-aortic balloon pumps (IABPs) and extracorporeal membrane oxygenation (ECMO), and incomplete data. Additionally, those exhibiting severe perioperative organ dysfunction and adverse events were excluded. A total of 75 patients were enrolled. Five patients were excluded from the final analysis: two required reoperation for postoperative bleeding, one required ECMO, one underwent an emergency cardiopulmonary bypass postoperatively, and one had severe pulmonary dysfunction. Thus, 70 patients were included in the final analysis.

Patients were divided into two groups based on their intraoperative urine output: the diuresis group (average urine output during surgery > 5 mL/kg/h, n = 38) and the normal group (n = 32) [9].

### 2.2. Intraoperative Management

All patients received general anesthesia and underwent standardized OPCABG procedures. Anesthesia induction was performed with Remimazolam, Etomidate, Rocuronium, and Sufentanil. Anesthetic maintenance consisted of Ciprofol, Dexmedetomidine, Rocuronium, and Sufentanil. Dopamine was administered for inotropic support, and norepinephrine was used to increase peripheral vascular resistance. Anesthetic management, fluid therapy, hemodynamic monitoring, and the use of vasoactive agents were guided by institutional protocols. Heparin was administered to achieve an activated clotting time (ACT) greater than 300 s. Heparin was neutralized with protamine sulfate in at a 1:1 ratio. All patients were transferred to the Cardiothoracic Surgery Intensive Care Unit after surgery.

### 2.3. Data Collection

Baseline demographics, anthropometric data, comorbidities (including hypertension and diabetes), preoperative laboratory values, fluid balance data, intraoperative hemodynamics, and postoperative recovery parameters were recorded. Hormonal and biochemical markers were assessed at five time points: T0 (preoperative baseline), T1 (end of surgery), T2 (24 h postoperatively), T3 (48 h postoperatively), and T4 (7 d postoperatively). It should be clarified that not all variables were assessed at the same perioperative time points. NT-proBNP and aldosterone were measured at T0, T1, and T2, whereas copeptin, electrolytes, glucose, lactate (Lac), and both plasma and urine osmolality were measured only at T0 and T1. Serum creatinine was evaluated at baseline (T0), 48 h postoperatively (T3), and 7 days postoperatively (T4). To ensure the accuracy of copeptin measurements, serum copeptin levels were determined using the Thermo Scientific BRAHMS Copeptin proAVP KRYPTOR assay (Thermo Scientific, Waltham, MA, USA).

### 2.4. Outcome Definitions

Primary outcome: occurrence of intraoperative high-volume diuresis (average urine output during surgery > 5 mL/kg/h) [9].

Secondary outcomes: temporal changes in biochemical markers, fluid balance, intraoperative hemodynamics, and postoperative recovery parameters.

### 2.5. Statistical Analysis

Missing data were minimal in this cohort and therefore were not imputed; analyses were performed on complete cases only. Analyses were performed using SPSS version 26.0 (IBM Corp., Armonk, NY, USA). The normality of continuous variables was assessed with the Shapiro–Wilk test. Normally distributed variables were expressed as the mean ± standard deviation (SD) and compared using the independent-samples *t* test. Non-normally distributed variables were presented as the median (interquartile range, IQR) and compared using the Mann–Whitney U test.

Categorical data were summarized as counts and percentages, and differences between groups were assessed with the χ^2^ test or Fisher’s exact test.

For within-group temporal comparisons, the Friedman test (non-parametric repeated measures) was applied. When significant, post hoc pairwise Wilcoxon signed-rank tests were conducted, with Bonferroni correction for multiple comparisons. Given the exploratory nature of this study, *p*-values should be interpreted as descriptive; adjustments were applied for repeated within-group comparisons. Between-group comparisons at each time point, as well as comparisons of Δ values, were performed using the Mann–Whitney U test.

To explore the factors associated with intraoperative high-volume diuresis, a binary logistic regression analysis with stepwise selection was performed. Variables with *p* < 0.10 in the univariate analysis were included in the candidate pool in order to avoid omitting potentially relevant predictors given the limited sample size, and clinically relevant variables were additionally considered for inclusion. Statistical significance in the final model was defined as *p* < 0.05. Results are reported as odds ratios (ORs) with corresponding 95% confidence intervals (CIs).

The predictive performance of individual predictors and combined models was evaluated using receiver operating characteristic (ROC) curve analysis, with the area under the curve (AUC) calculated for discrimination. To minimize overfitting, the number of variables in the final model was restricted according to the events per variable (EPV) principle (EPV > 10). Internal validation via bootstrap resampling was planned, but this could not be performed due to software limitations. The relatively high EPV (>10) reduced the likelihood of overfitting, although external validation remains necessary.

All statistical tests were two-sided, and a *p* value < 0.05 was considered to indicate statistical significance.

## 3. Results

### 3.1. Baseline Characteristics

The baseline demographic and clinical characteristics of patients in the normal (n = 32) and diuresis (n = 38) groups are presented in Table 1.

Patients in the diuresis group were significantly older than those in the normal group (63.7 ± 6.2 vs. 58.2 ± 9.9 years, t = −2.814, *p* = 0.006). Height, weight, and BMI also differed between groups, with the diuresis group having lower values (height: 163.5 ± 7.5 vs. 167.3 ± 7.1 cm, t = 2.141, *p* = 0.036; weight: 66.3 ± 9.9 vs. 78.1 ± 10.2 kg, t = 4.871, *p* < 0.001; BMI: 24.8 ± 3.4 vs. 27.9 ± 3.2 kg/m^2^, t = 3.919, *p* < 0.001).

There was no statistically significant difference in sex distribution between groups (male: 63.2% vs. 84.4%, *p* = 0.061). Regarding comorbidities, the prevalence of hypertension (47.4% vs. 50.0%, *p* = 0.826) and diabetes mellitus (34.2% vs. 28.1%, *p* = 0.585) did not differ significantly between groups.

Preoperative admission vital signs did not differ significantly between groups. Regarding laboratory parameters, preoperative serum creatinine was slightly lower in the diuresis group (64.7 [55.85–70.7] vs. 68.4 [62.55–78.35] µmol/L, *p* = 0.027), urine osmolality, and copeptin also showed significant differences (T0 urine osmolality: 519.2 ± 246.4 vs. 626.7 ± 163.6 mOsm/kg, t = 2.177, *p* = 0.033; T0 copeptin: 4.25 [2.31–7.67] vs. 6.02 [4.21–10.05] pmol/L, U = 413.5, *p* = 0.022). Other parameters were not significantly different between groups.

Intraoperative urine output was markedly higher in the diuresis group (2000 [1875–3000] vs. 900 [650–1200] mL, U = 588, *p* < 0.001).

Overall, the groups were comparable in terms of most baseline characteristics, with the exception of age, anthropometric measures, preoperative serum creatinine, T0 urine osmolality, and T0 copeptin.

### 3.2. Binary Logistic Regression Analysis of Factors Associated with Diuresis

**Figure 1:**
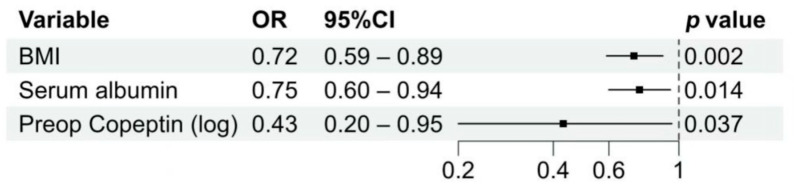
Independent predictors of perioperative diuresis identified by multivariable logistic regression. Odds ratios (ORs) with 95% confidence intervals (CIs) are shown for the three independent predictors of intraoperative high-volume diuresis. Lower body mass index (BMI), reduced preoperative serum albumin, and lower log-transformed preoperative copeptin were significantly associated with an increased risk of intraoperative high-volume diuresis. Squares represent point estimates of the OR, and horizontal lines indicate 95% CIs.

In the final model obtained from the stepwise logistic regression analysis (Table 2), preoperative log-transformed copeptin (LNT0Copeptin) (OR = 0.43, 95% CI 0.20–0.95, *p* = 0.037), preoperative albumin level (OR = 0.75, 95% CI 0.60–0.94, *p* = 0.014), and BMI (OR = 0.72, 95% CI 0.59–0.89, *p* = 0.002) were identified as independent predictors of diuresis (Figure 1).

The model also included potential covariates such as hypertension, diabetes, eGFR, preoperative serum creatinine, preoperative BUN, ΔCopeptin, T0 aldosterone, T0 NT-proBNP, age, and sex, but these factors were not significantly associated with diuresis in the final model (*p* > 0.05).

**Figure 2:**
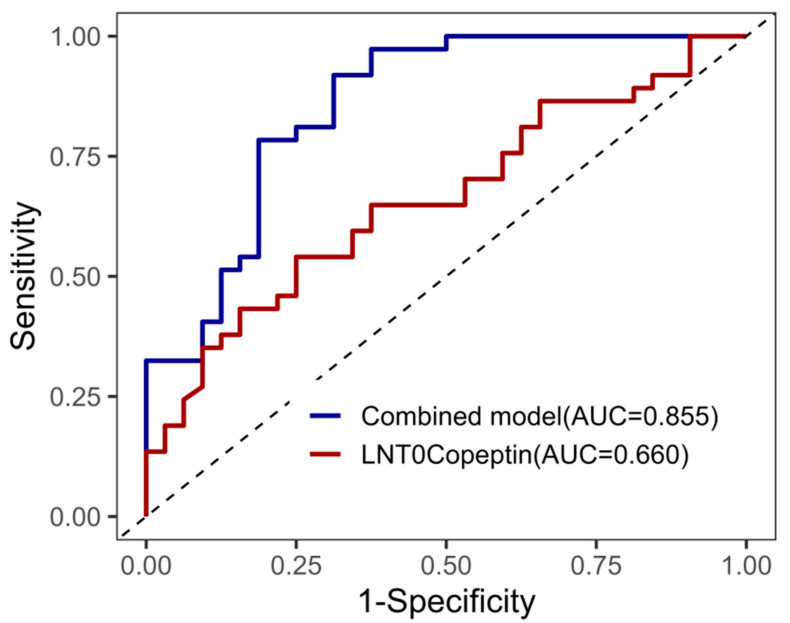
ROC curves for prediction of diuresis. ROC = Receiver Operating Characteristic; AUC = Area Under the Curve. The combined model includes BMI, preoperative albumin, and LNT0Copeptin.The dashed diagonal line represents the line of no discrimination (AUC = 0.5), indicating the performance of a non-informative test.

As shown in Figure 2, an ROC curve analysis was performed to evaluate the predictive performance of LNT0Copeptin and the combined model for diuresis. The combined model, incorporating BMI, preoperative albumin, and LNT0Copeptin, achieved an area under the curve (AUC) of 0.855, indicating a good discriminative ability. In comparison, LNT0Copeptin alone yielded an AUC of 0.660, suggesting moderate predictive performance.

### 3.3. Plasma Sodium, Perioperative Hormonal Activity and Osmolality

At baseline (T0), plasma osmolality and serum sodium were comparable between the two groups (both *p* > 0.05), whereas urine osmolality was significantly higher in the group with normal urine output (626.66 ± 163.60 vs. 519.24 ± 246.42 mOsm/kg, *p* = 0.039). At the end of surgery (T1), patients with intraoperative high-volume diuresis exhibited significantly higher plasma osmolality (313.0 [308.7–317.2] vs. 306.0 [303.0–310.0] mOsm/kg, *p* < 0.001) and serum sodium concentrations (148.42 ± 3.33 vs. 144.94 ± 2.69 mmol/L, *p* < 0.001), while urine osmolality became similar between the groups (*p* = 0.708). The analysis of Δ (T1–T0) revealed a significantly greater increase in plasma osmolality (Z = −2.830, *p* = 0.005) and serum sodium (t = −5.389, *p* < 0.001) in the diuresis group. Although urine osmolality tended to decrease more markedly in the normal group, the between-group difference was not statistically significant (t = −1.811, *p* = 0.075) (Table 3).

### 3.4. Perioperative Hormonal Changes

#### 3.4.1. Comparison of Biomarkers Between Diuresis and Normal Groups

At baseline (T0), there were no significant differences in NT-proBNP or aldosterone levels between the diuresis and normal groups (all *p* > 0.1). The T0 copeptin levels were significantly lower in the diuresis group than in the normal group (median [IQR]: 4.24 [2.31–7.67] vs. 6.03 [4.22–10.04] pmol/L, *p* = 0.022). At T1 and T2, there were no statistically significant differences between the groups regarding for NT-proBNP, aldosterone, or copeptin (all *p* > 0.05). The changes (Δ) from baseline (T0) to T1 or T2 also did not differ significantly between groups (all *p* > 0.05) (Table 4).

#### 3.4.2. Temporal Changes in NT-proBNP, Aldosterone and Copeptin

**Figure 3:**
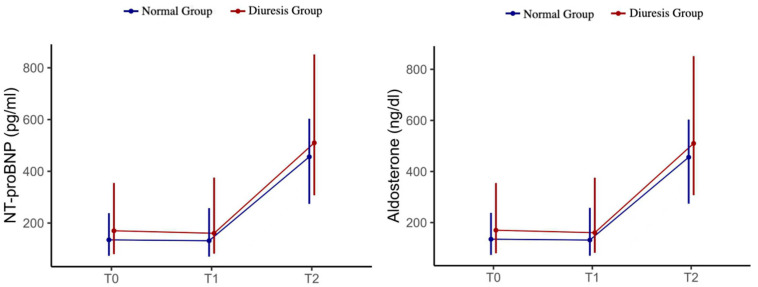
Temporal changes in NT-proBNP and aldosterone between diuresis and normal groups. NT-proBNP and aldosterone were measured at baseline (T0), end of surgery (T1), and 24 h postoperatively (T2). NT-proBNP increased significantly over time in both groups (Friedman test, *p* < 0.001), while aldosterone levels showed a significant increase only in the diuresis group (*p* = 0.04), with no significant change in the normal group (*p* = 0.19). Data are presented as median (interquartile range). Error bars represent interquartile ranges.

Within-group analysis showed that NT-proBNP increased significantly over time in both groups (Friedman test: χ^2^ = 37.75, *p* < 0.001 for normal group; χ^2^ = 34.28, *p* < 0.001 for diuresis). Pairwise comparisons by Post hoc Wilcoxon tests demonstrated significant increases from T0 to T2 and from T1 to T2 in both groups (Bonferroni-corrected *p* < 0.001). Aldosterone levels increased over time in the diuresis group (χ^2^ = 6.463, *p* = 0.04), but not in the normal group (χ^2^ = 3.323, *p* = 0.19). Post hoc Wilcoxon tests for aldosterone in the diuresis group showed a borderline increase from T1 to T2 (*p* = 0.087, Bonferroni-corrected). These temporal trends are illustrated in Figure 3, which shows the rise in NT-proBNP in both groups and the selective increase in aldosterone in the diuresis group.

Copeptin levels showed no significant temporal changes in either group. In the diuresis group, copeptin increased slightly from T0 to T1, but this was not statistically significant (*p* = 0.5). In the normal group, copeptin remained stable from T0 to T1 (*p* = 0.34) (Table 5).

### 3.5. Perioperative Changes in Creatinine

Perioperative changes in serum creatinine showed slight differences between groups; these changes reached statistical significance, although the overall clinical impact appeared minimal. Detailed statistics are provided in Table 6 and Table 7.

### 3.6. Impact of Intraoperative High-Volume Diuresis on Prognosis

Perioperative hemodynamic and biochemical parameters, intraoperative management, and clinical outcomes were compared between the normal and diuresis groups (Table 8).

At the end of surgery, there were no significant differences in systolic blood pressure, diastolic blood pressure, heart rate, lactate, and glucose levels or serum potassium between the two groups (all *p* > 0.05). However, serum sodium was significantly higher in the diuresis group than in the normal group (148.42 ± 3.33 vs. 144.94 ± 2.69 mmol/L, mean difference = −3.48, 95% CI: −4.92 to −2.05, *p* < 0.001).

Regarding intraoperative management, the dopamine and norepinephrine infusion rates, operation time, and blood loss did not differ significantly between the two groups (all *p* > 0.05). In contrast, the total fluid infusion volume was significantly higher in the diuresis group than in the normal group (2750 [2350–3250] vs. 2100 [2000–2450] mL, U = 253.5, *p* < 0.001).

Urine output during surgery was also markedly greater in the diuresis group (2000 [1900–3000] vs. 900 [700–1200] mL, U = 55.0, *p* < 0.001). Postoperative outcomes, including the ICU stay and total hospital stay, were comparable between the two groups (all *p* > 0.05).

In summary, patients with intraoperative high-volume diuresis exhibited higher intraoperative sodium levels, received larger volumes of fluid infusion, and produced significantly greater urine output than normal patients, whereas other hemodynamic, biochemical, and outcome parameters were similar.

## 4. Discussion

A total of 70 patients were included in the final analysis; of these, 38 experienced intraoperative high-volume diuresis, corresponding to an incidence of 54.2%, which is notably higher than that reported in other types of surgery. Diuresis is a complex physiological phenomenon involving multiple stimuli and mechanisms. To further explore the underlying causes of high urine output during OPCABG, it is essential to first characterize the type of diuresis based on the measured osmotic parameters and subsequently analyze the contributing factors according to relevant biochemical and hormonal changes.

### 4.1. Classification of Diuresis

In this study, urine osmolality remained above 300 mOsm/kg at both T0 and T1 in the diuresis group and in the normal group, indicating the presence of an osmotic component in the intraoperative diuresis [9]. However, during OPCABG, we observed a continuous increase in plasma osmolality and serum sodium levels, with more pronounced elevations in the diuresis group. Meanwhile, higher urine output was associated with lower urine osmolality, approaching approximately 300 mOsm/kg and showing a minimal concentrating ability. This pattern suggests that the high urine output during surgery resulted predominantly from excessive electrolyte-free water loss, with relatively limited solute excretion and a certain degree of impairment in renal concentrating capacity. Therefore, it is assumed that the intraoperative diuresis observed in this study represented a mixed form of osmotic diuresis and water diuresis.

#### 4.1.1. Osmotic Diuresis

In this study, blood glucose and lactate levels at both T0 and T1 were within normal ranges and showed no significant differences between groups, ruling out osmotic diuresis driven by glucose or lactate excretion.

Previous studies have suggested that excessive fluid administration may disrupt the renal medullary osmotic gradient, reduce urea reabsorption, and consequently contribute to osmotic diuresis. However, in this study, a relatively restrictive fluid strategy was employed during OPCABG to avoid volume overload. Despite the similar intraoperative blood loss between groups, urine output in the diuresis group was approximately 1000 mL higher, whereas only around 500 mL more fluid was administered. This discrepancy indicates substantially greater free-water loss in the diuresis group than could be explained by fluid loading alone; thus, this mechanism was unlikely to be a major determinant of the high-volume diuresis observed.

Although elevated BNP may contribute to osmotic diuresis—given that cardiac traction and hemodynamic fluctuations during cardiac surgery can activate the natriuretic peptide system and promote sodium excretion—in this study, BNP levels did not show a substantial increase immediately after surgery, nor did they differ significantly between groups. These findings suggest that BNP was unlikely to have been a major determinant of the intraoperative high urine output observed [10].

Taken together, while an osmotic component contributed to the increased urine output, osmotic diuresis alone could not account for the substantial difference in urine output between groups. The overall pattern is best explained by mixed diuresis, in which water diuresis predominates, accompanied by a minor degree of solute-driven natriuresis.

#### 4.1.2. Water Diuresis

Water diuresis can be categorized into central diabetes insipidus (CDI), nephrogenic diabetes insipidus (NDI), primary polydipsia (PP), and secondary or transient water diuresis induced by perioperative factors [11,12]. Copeptin is the stable C-terminal fragment of the vasopressin precursor; it is a reliable surrogate biomarker of Arginine Vasopressin (AVP) and significantly improves the differential diagnosis of CDI, NDI and PP [13].

PP typically occurs in conscious patients and is driven by excessive behavioral water intake. Given that all subjects in this study were under general anesthesia, with fluid administration strictly controlled by clinicians, PP could be reasonably be excluded as the primary cause of intraoperative diuresis.

In anesthetized patients, the infusion of large-volume crystalloid solutions infusion may cause blood volume expansion, suppress Renin–Angiotensin–Aldosterone System (RAAS) and sympathetic activity, and enhance natriuretic peptide release, thereby reducing tubular sodium reabsorption. Concurrently, decreased plasma osmolality suppresses AVP release, leading to reduced free water reabsorption and increased urine output with diluted urine. However, in the present study, despite comparable intraoperative blood loss, the diuresis group produced approximately 1000 mL more urine while receiving only about 500 mL more fluid than the control group, indicating a net excess of free water loss. Additionally, diuresis was accompanied by increases in plasma sodium and osmolality, which contradicts the dilutional pattern typically associated with hypotonic infusion–induced water diuresis. These findings suggest that volume expansion–related water diuresis alone is unlikely to fully explain the marked intraoperative urine output observed.

Basal copeptin levels ≥21.6 pmol/L strongly indicate NDI; however, the copeptin concentrations in both groups were markedly below this diagnostic threshold, suggesting the absence of vasopressin resistance and enabling the exclusion of NDI as the underlying mechanism.

At the beginning of surgery, baseline copeptin in the diuresis group was 4.6 pmol/L, falling within the intermediate diagnostic range of 2.6–21.4 pmol/L [14,15,16], which allowed us to rule out complete CDI but implied a potential partial impairment in AVP secretion. Although a standardized hypertonic saline stimulation test was not performed, plasma osmolality and sodium levels significantly increased during the operation, with plasma sodium approaching 148 mmol/L, which is comparable to the osmotic activation threshold applied in hypertonic testing protocols. Under this hyperosmolar condition, copeptin increased only modestly from 4.6 to 5.2 pmol/L, slightly exceeding the 4.9 pmol/L cut-off but without statistical significance, indicating a blunted osmotic response. Therefore, the partial CDI-like suppression of vasopressin secretion during surgery could not be excluded.

In conclusion, our findings indicate that the high-volume diuresis occurring during OPCABG is best classified as mixed diuresis—primarily water diuresis with a minor osmotic component—consistent with impaired AVP-mediated concentrating responses.

### 4.2. Hormones and Diuresis

#### 4.2.1. Copeptin and ADH

The logistic regression analysis of the baseline characteristics revealed that a lower BMI and lower serum albumin and copeptin levels were independently associated with intraoperative high-volume diuresis. Given that copeptin reflects endogenous AVP secretion, a reduction in copeptin strongly suggests impaired vasopressin-mediated water regulation as a potential driving mechanism [17,18]. These findings not only corroborate our classification of the diuresis phenotype but also support the involvement of a partial CDI-like AVP deficiency in the development of intraoperative high-volume diuresis. Thus, intraoperative high-volume diuresis appears not to be a random occurrence but rather a manifestation of disrupted neuroendocrine and fluid homeostasis during surgery.

#### 4.2.2. NT-proBNP and Aldosterone Response

The increase in NT-proBNP over time in both groups highlights a common physiological response to the surgical stress and volume shifts that occur during OPCABG. NT-proBNP is an established biomarker for heart failure and is indicative of myocardial stretch, reflecting the increased workload of the heart; it plays an important role in cardiorenal protection [10,19,20]. Although NT-proBNP increased significantly in both groups, no significant difference was observed between the groups, suggesting that cardiac stress may not have been the main determinant of diuresis in this cohort.

The baseline aldosterone levels did not differ significantly between the diuresis and normal groups. However, over time, the aldosterone levels increased significantly in the diuresis group, while no significant change was observed in the normal group, with a borderline increase from T1 to T2. This trend suggests that aldosterone is not directly associated with the occurrence of diuresis. However, in response to perioperative fluid and electrolyte shifts, the renin–angiotensin–aldosterone system (RAAS) may be activated as a compensatory mechanism in those with diuresis. Given the central role of aldosterone in sodium and water reabsorption [21,22], this compensatory mechanism may have contributed to the postoperative normalization of the fluid balance in the diuresis group.

### 4.3. Effects of Intraoperative Medications on Diuresis

In addition to osmotic and AVP-related mechanisms, perioperative medications may also influence urine production. Dopamine [3], which is often used for hemodynamic support during OPCABG, exerts diuretic and natriuretic effects through dopaminergic receptor activation in the renal proximal tubule, thereby inhibiting sodium reabsorption and suppressing both AVP release and its renal actions. This results in water diuresis with a natriuretic component. In this study, although dopamine administration was limited and comparable between groups, the contribution of its physiological effects to the increased urine output observed cannot be excluded.

Dexmedetomidine has also been reported [3], albeit rarely, to cause transient diabetes insipidus-like water diuresis through the α2-adrenergic suppression of hypothalamic AVP release. The mechanism appears to be independent of hemodynamic changes, but whether it is dose-dependent remains unclear. In our cohort, dexmedetomidine was administered following standardized anesthetic protocols with similar dosing between groups; thus, it was unlikely to be the primary cause of the observed high-volume diuresis. Nevertheless, the potential interaction between sedative agents and the neuroendocrine regulation of fluid balance warrants further investigation.

Collectively, although pharmacologic influences cannot be completely ruled out, their impact in this study appears limited, and the observed diuresis was more likely to related to altered osmotic and AVP-mediated water regulation.

### 4.4. Surgery-Specific Features of Intraoperative Diuresis

Perioperative disturbances in water balance exhibit marked heterogeneity across different surgical types. Unlike the mixed pattern of diuresis observed in the OPCABG patients in our study—characterized predominantly by water diuresis with hyperosmolar features—neurosurgical and trans-sphenoidal pituitary procedures are more commonly associated with oliguria and hypotonic hyponatremia due to excessive AVP secretion, presenting a typical SIADH-like profile [23]. In thoracic surgeries involving extensive pulmonary manipulation, both intraoperative polyuria and postoperative brisk natriuresis with hyponatremia have been reported [1,24]. These discrepancies indicate that the direction and magnitude of perioperative fluid imbalance are strongly influenced by the type of surgery and the pattern of neuroendocrine activation involved.

The prominent polyuric response during OPCABG may be related to cardiac traction, altered venous return, and stress on the atria and great vessels, which may suppress AVP secretion while promoting BNP release, thereby generating a pathophysiological pattern opposite to SIADH. Thus, the polyuria observed in this study likely reflects a procedure-specific disturbance in fluid regulation that is uniquely associated with OPCABG.

Therefore, intraoperative polyuria is not a universal perioperative phenomenon but appears to be particularly relevant to cardiac surgery, especially beating-heart coronary artery bypass grafting. Whether this response is unique to OPCABG or also present in other forms of cardiac surgery requires further confirmation through larger multicenter studies.

### 4.5. Clinical Implications

In our study, intraoperative high-volume diuresis was characterized by consistently lower serum creatinine levels across all time points in comparison to normal patients, suggesting that it was not associated with renal impairment but instead reflected preserved or even enhanced glomerular filtration. Both groups exhibited significant temporal declines in creatinine, further indicating an overall postoperative improvement in renal function. Notably, although diuresis patients exhibited higher serum sodium levels and significantly increased intraoperative urine output, these changes did not lead to hemodynamic instability, significant biochemical disturbances, or prolonged ICU and hospital stays. These results suggest that intraoperative high-volume diuresis is not a marker of poor prognosis.

However, diuresis did present challenges in intraoperative fluid management, as patients required significantly more fluid infusion to maintain circulatory stability. In our cohort, adequate and timely volume replacement effectively prevented adverse outcomes. Nevertheless, this phenomenon also emphasizes the need for the careful monitoring of electrolyte homeostasis, particularly serum sodium levels, in patients with diuresis [25]. We observed an association between diuresis and mild hypernatremia, which suggests that even in the absence of overt renal dysfunction, improper fluid management may still lead to electrolyte disturbances.

Although intraoperative high-volume diuresis was not associated with adverse short-term outcomes in our cohort, this finding should be interpreted with caution. Given the relatively small sample size and single-center design, the possibility of β error (insufficient statistical power) cannot be excluded. Therefore, larger multicenter studies are warranted to confirm that intraoperative high-volume diuresis truly has no impact on prognosis.

### 4.6. Limitations

This study has several limitations. Firstly, it was a single-center observational cohort study with a relatively small sample size, which may limit the generalizability of the findings and raises the possibility of β errors due to limited statistical power. Secondly, although key hormonal markers were measured, the number of perioperative assessment time points was limited, which may have prevented us from capturing the full dynamic changes in these hormones. Thirdly, postoperative urine output was not continuously monitored, restricting our ability to characterize the temporal recovery of diuresis in detail. Finally, hypertonic saline stimulation, water deprivation, and desmopressin testing were not performed; therefore, the diagnostic certainty regarding partial central diabetes insipidus could not be established, and the current results should be regarded as hypothesis-generating only. Larger, multicenter studies with more comprehensive perioperative measurements are warranted to validate and extend our findings.

## 5. Conclusions

Although intraoperative high-volume diuresis did not adversely affect patient outcomes in our study, it poses considerable challenges for anesthesiologists in intraoperative management. Patients with excessive urine output required substantially larger volumes of intravenous fluid to maintain adequate circulating volumes, as well as more refined hemodynamic monitoring to avoid episodes of hypotension and prevent electrolyte disturbances such as hypernatremia. This additional burden highlights that intraoperative high-volume diuresis, while not directly detrimental to short-term outcomes, nevertheless complicates intraoperative care and necessitates individualized fluid and electrolyte management strategies.

Future studies with larger sample sizes and multicenter designs are warranted to confirm these findings and to further clarify the clinical implications of intraoperative high-volume diuresis, including its potential impact on long-term renal and cardiovascular outcomes. In addition, mechanistic investigations are needed to determine whether impaired antidiuretic hormone (ADH) secretion, partial central diabetes insipidus, and renal tubular stress are predominant drivers of this phenomenon. Pharmacological interventions, such as the use of vasopressin or posterior pituitary extracts, may provide valuable therapeutic options [26]. Given their ability to regulate both vascular tone and water homeostasis, these agents could help to reduce urine output, stabilize blood pressure, and mitigate electrolyte imbalances, thereby offering a targeted strategy for patients at high risk of perioperative or intraoperative high-volume diuresis.

## Figures and Tables

**Table 1:** Baseline characteristics between normal and diuresis groups.

Variable	Normal (n = 32)	Diuresis (n = 38)	Mean Difference (95% CI)	t Value	*p*-Value	U Value
Age (years)	58.19 ± 9.90	63.66 ± 6.21	−5.47 (−9.35, −1.59)	−2.814	0.006	–
Height (cm)	167.25 ± 7.12	163.47 ± 7.54	3.78 (0.26, 7.30)	2.141	0.036	–
Weight (kg)	78.06 ± 10.20	66.33 ± 9.89	11.73 (6.92, 16.53)	4.871	<0.001	–
BMI (kg/m^2^)	27.91 ± 3.19	24.82 ± 3.36	3.09 (1.52, 4.66)	3.919	<0.001	–
Male sex, n (%)	27 (84.4)	24 (63.2)	–	–	0.061	–
Hypertension, n (%)	16 (50.0)	18 (47.4)	–	–	0.826	–
Diabetes, n (%)	9 (28.1)	13 (34.2)	–	–	0.585	–
EF (%)	63.0 [58.0–65.5]	62.0 [59.0–66.0]	–	–	0.859	–
eGFR (mL/min/1.73 m^2^)	94.03 [88.03–100.01]	94.59 [91.0–99.8]	–	–	0.625	–
Intraoperative hypothermia, %	28.1	47.4	–	–	0.140	–
Albumin (g/L)	42.0 [39.65–45.2]	41.75 [39.0–42.9]	–	–	0.082	–
Preop Cr (µmol/L)	68.4 [62.55–78.35]	64.7 [55.85–70.7]	–	–	0.027	–
Preop BUN (mmol/L)	6.32 ± 1.16	6.07 ± 1.39	0.25 (−0.40, 0.90)	0.771	0.444	–
Admission SBP (mmHg)	166.1 ± 28.8	167.6 ± 19.0	−1.43 (−13.37, 10.52)	−0.240	0.811	–
Admission DBP (mmHg)	78.6 ± 12.4	75.7 ± 9.3	2.83 (−2.35, 7.99)	1.090	0.279	–
Admission HR (beats/min)	70 [63–80.75]	65 [60.75–74.25]	–	–	0.160	489
T0 Potassium (mmol/L)	3.58 ± 0.32	3.51 ± 0.34	0.08(−0.09, 0.23)	0.904	0.370	–
T0 Sodium (mmol/L)	143.0 [142.0–144.0]	143.0 [142.0–145.0]	–	–	0.296	521
T0 Lactate (mmol/L)	0.80 [0.60–1.10]	0.70 [0.60–0.93]	–	–	0.548	557.5
T0 Glucose (mmol/L)	6.20 [5.43–7.18]	5.80 [5.28–6.58]	–	–	0.280	516.5
T0 urine osmolality (mOsm/kg)	626.66 ± 163.60	519.24 ± 246.42	107.4 (8.9, 206.0)	2.177	0.033	–
T0 plasma osmolality (mOsm/kg)	297 [294–300]	298 [295–302]	–	–	0.327	525
T0 Copeptin (pmol/L)	6.02 [4.21–10.05]	4.25 [2.31–7.67]	–	–	0.022	413.5
T0 Aldosterone (ng/dL)	4.0 [3.13–6.30]	3.75 [3.10–4.95]	–	–	0.457	545
T0 NT-proBNP (pg/mL)	135.0 [71.8–239.4]	154.3 [73.0–358.2]	–	–	0.759	582
Intraoperative urine output (mL)	900 [650–1200]	2000 [1875–3000]	–	–	<0.001	588
ΔCopeptin (pmol/L)	0.058 [−1.41, 3.97]	0.093 [−1.69, 0.93]	–	–	0.814	55

Data are presented as mean ± SD, median [IQR], or n (%). Statistical comparisons were performed using an independent *t* test for normally distributed continuous variables, the Mann–Whitney U test for non-normally distributed continuous variables, and the χ^2^ test for categorical variables. Preop Cr = Preoperative Serum Creatinine; Preop BUN= Preoperative Blood Urea Nitrogen; SBP = Systolic Blood Pressure; DBP = Diastolic Blood Pressure; HR = Heart Rate.

**Table 2:** Stepwise Binary Logistic Regression Analysis for diuresis (Step 3, Final Model).

Variable	B	SE	Wald	df	*p*	OR	95% CI for OR
LNT0Copeptin	−0.835	0.400	4.348	1	0.037	0.434	0.198–0.951
Albumin (g/L)	−0.284	0.115	6.054	1	0.014	0.753	0.600–0.944
BMI	−0.325	0.104	9.865	1	0.002	0.722	0.589–0.885
Hypertension (1)	0.296	–	0.296	1	0.587	–	–
Diabetes (1)	0.042	–	0.042	1	0.838	–	–
eGFR (mL/min/1.73 m^2^)	−0.486	–	0.486	1	0.486	–	–
Preop Cr (µmol/L)	−1.221	–	1.221	1	0.269	–	–
Preop BUN (mmol/L)	−0.455	–	0.455	1	0.500	–	–
ΔCopeptin (pmol/L)	−0.199	–	0.199	1	0.655	–	–
T0 aldosterone (ng/dL)	−0.322	–	0.322	1	0.571	–	–
T0 NT-proBNP (pg/mL)	−0.914	–	0.914	1	0.339	–	–
Age	−0.386	–	0.386	1	0.534	–	–
Sex (1 = male)	−1.518	–	1.518	1	0.218	–	–
Constant	21.975	6.584	11.139	1	0.001	3.50 × 10^9^	–

1. Subsubsec: B = regression coefficient; SE = standard error; OR = odds ratio; CI = confidence interval; Wald = Wald test; df = degrees of freedom. 2. Step 3 represents the final model, including both significant and non-significant covariates. 3. OR < 1 indicates that an increase in the variable is associated with a decreased likelihood of diuresis.

**Table 3:** Plasma and Urine Osmolality in Diuresis and Normal Groups.

Variable	Time Point	Normal	Diuresis	t/Z	*p*-Value
Plasma osmolality(mOsm/kg)	T0	297.0 [294.0–300.0]	298.0 [294.7–302.0]	−0.981	0.327
	T1	306.0 [303.0–310.0]	313.0 [308.7–317.2]	−3.836	< 0.001
	Δ (T1–T0)	10.0 [5.0–12.0]	16.0 [10.0–19.3]	−2.830	0.005
Urine osmolality (mOsm/kg)	T0	626.66 ± 163.60	519.24 ± 246.42	2.105	0.039
	T1	332.72 ± 90.24	324.24 ± 95.81	0.376	0.708
	Δ (T1–T0)	−293.94 ± 178.51	−191.30 ± 274.04	−1.811	0.075
Plasma Sodium (mmol/L)	T0	142.91 ± 1.89	143.50 ± 2.32	−1.159	0.251
	T1	144.94 ± 2.69	148.42 ± 3.33	−4.758	<0.001
	Δ (T1–T0)	2.03 ± 2.33	4.92 ± 2.15	−5.389	*p* < 0.001

Plasma osmolality and plasma sodium are presented as mean ± SD, and urine osmolality is presented as median (interquartile range). Group comparisons were performed using an independent *t*-test or the Mann–Whitney U test.

**Table 4:** Comparison of biomarkers between diuresis and normal groups (Mann–Whitney U test).

Variable	Time Point	Normal Median (IQR)	Diuresis Median (IQR)	Z/*p*-Value
NT-proBNP (pg/mL)	T0	135.02 [73.47–238.16]	170.17 [80.13–354.92]	−0.307/0.759
	T1	131.76 [70.52–257.56]	160.52 [81.88–375.82]	−0.393/0.694
	T2	455.85 [274.17–603.19]	509.94 [307.38–851.57]	−1.307/0.300
	Δ (T1–T0)	−8.73 [−25.13, 7.67]	−16.64 [−44.8, −1.93]	−1.149/0.251
	Δ (T2–T0)	261.86 [154.86–432.24]	240.79 [119.87–663.07]	−0.259/0.795
	Δ (T2–T1)	303.66 [144.81–451.01]	266.42 [133.00–716.17]	−0.184/0.854
Aldosterone (ng/dL)	T0	4.0 [3.1–6.3]	3.8 [2.8–4.7]	−0.743/0.457
	T1	3.6 [2.7–5.3]	3.1 [2.1–4.1]	−1.337/0.181
	T2	4.6 [3.1–6.4]	3.8 [3.0–5.5]	−0.307/0.102
	Δ (T1–T0)	−0.75 [−2.20–0.38]	−0.80 [−1.70–0.93]	−0.226/0.821
	Δ (T2–T0)	0.65 [−0.58–1.93]	−0.05 [−1.33–2.30]	−0.737/0.461
	Δ (T2–T1)	1.00 [−1.10–3.22]	1.20 [−0.75–3.33]	−0.226/0.821
Copeptin (pmol/L)	T0	6.03 [4.22–10.04]	4.24 [2.31–7.67]	−2.293/0.022
	T1	7.65 [4.78–13.62]	5.74 [3.23–9.52]	−1.651/0.099
	Δ (T1–T0)	0.06 [−1.41–3.97]	−0.48 [−1.69–3.46]	−0.236/0.814

Bulleted lists look like tValues are presented as median (interquartile range). Comparisons between diuresis and normal groups were performed using the Mann–Whitney U test.

**Table 5:** Changes in BNP and Aldosterone over Time in Diuresis and Normal Groups according to Pairwise Comparison of Biomarkers Between Two Time Points (Wilcoxon Signed-Rank Test).

Variable	Group	Time Points	Z	*p* (Uncorrected)	*p* (Bonferroni Corrected)
NT-proBNP (pg/mL)	Normal (n = 32)	T0 vs. T1	−1.627	0.104	0.312
		T0 vs. T2	4.824	<0.001	<0.001
		T1 vs. T2	4.824	<0.001	<0.001
	Diuresis (n = 38)	T0 vs. T1	−2.812	0.005	0.015
		T0 vs. T2	4.343	<0.001	<0.001
		T1 vs. T2	4.766	<0.001	<0.001
Aldosterone (ng/dL)	Diuresis (n = 38)	T0 vs. T1	−1.654	0.098	0.294
		T0 vs. T2	0.347	0.729	1
		T1 vs. T2	2.189	0.029	0.087
Copeptin (pmol/L)	Normal (n = 32)	T0 vs. T1	0.954	0.34	
	Diuresis (n = 38)	T0 vs. T1	0.674	0.5	

Note. Data are presented as test statistics from the Wilcoxon signed-rank test. Bonferroni correction was applied for multiple pairwise comparisons. Post hoc pairwise Wilcoxon tests were only performed when the overall Friedman test indicated statistical significance.

**Table 6:** Groups Perioperative Creatinine: Between-Group Comparison.

Time Point	Group	Median [IQR]	Mann–Whitney U	Z	*p*-Value
Pre-op Cr (µmol/L)	Normal	68.4 [62.55–78.35]	420.5	−2.211	0.027
	Diuresis	64.7 [55.85–70.7]			
Post-op 48 h Cr (µmol/L)	Normal	75.0 [60.05–82.65]	374.5	−2.617	0.009
	Diuresis	63.45 [54.15–71.1]			
Post-op 7 d Cr (µmol/L)	Normal	65.0 [56.0–76.0]	416	−2.118	0.034
	Diuresis	54.7 [45.95–67.3]			

Notes. Data are presented as median [interquartile range]. Mann–Whitney U test was used for group comparisons at each time point. Postop = postoperative.

**Table 7:** Perioperative Creatinine: Within-Group Changes.

Variable	Group	Friedman χ^2^	*p*-Value	Pre-Opvs 48 h *p* (Bonferroni Corrected)	Pre-Op vs. 7 d *p* (Bonferroni Corrected)	48 h vs. 7 d *p* (Bonferroni Corrected)
Cr (µmol/L)	Normal	6.913	0.032	0.336	0.474	0.003
	Diuresis	6	0.05	1.000	0.018	0.018

Friedman test was used to assess within-group changes over time. Bonferroni correction was applied for post hoc pairwise comparisons.

**Table 8:** Perioperative Hemodynamic, Biochemical, and Clinical Parameters at end of Surgery in Normal and Diuresis Groups.

Variable	Normal (n = 32)	Diuresis (n = 38)	Mean Difference (95% CI)	t/U	*p*-Value
T1 SBP (mmHg)	122.88 ± 11.46	127.50 ± 12.69	−4.63 (−10.39, 1.14)	−1.59	0.114
T1 DBP (mmHg)	64.16 ± 7.28	65.47 ± 8.96	−1.32 (−5.19, 2.56)	−0.68	0.5
T1 heart rate (rate)	74.00 ± 10.56	71.71 ± 11.01	2.29 (−2.87, 7.45)	0.89	0.379
T1 potassium (mmol/L)	4.16 ± 0.43	4.34 ± 0.43	−0.18 (−0.38, 0.03)	−1.73	0.088
T1 sodium (mmol/L)	145.0 [143.0–147.0]	148.42 ± 3.33	−3.48 (−4.92, −2.05)	−4.85	<0.001
Dopamine (µg/kg/min)	3.00 (0.25–3.00)	2.00 (0.00–3.00)		530	0.336
Norepinephrine (µg/kg/min)	0.00 (0.00–0.00)	0.00 (0.00–0.00)		528.5	0.107
Operation time (h)	4.50 (4.00–5.25)	4.50 (4.00–5.00)		57.5	0.688
Blood loss (mL)	550 (500–650)	600 (500–600)		607	0.99
Total fluid infusion (mL)	2100 (2000–2450)	2750 (2350–3250)		253.5	<0.001
Urine output (mL)	900 (700–1200)	2000 (1900–3000)		55.0	<0.001
Lactate at T1 (mmol/L)	0.70 (0.60–1.00)	0.70 (0.60–0.90)		575	0.693
Glucose at T1 (mmol/L)	7.95 (6.40–9.50)	7.60 (6.60–9.10)		591.5	0.846
ICU stay (days)	1.00 (1.00–2.00)	1.00 (1.00–2.00)		560.5	0.51
Hospital stay (days)	16.0 (14.5–18.0)	17.0 (15.0–19.0)		478	0.123

Data are presented as mean ± SD or median (interquartile range) depending on distribution. Continuous variables with normal distribution were compared using independent *t*-test; non-normally distributed variables were compared using Mann–Whitney U test. BP, blood pressure; ICU, intensive care unit; T1, at the end of surgery.

## Data Availability

The raw data supporting the conclusions of this article will be made available by the authors, without undue reservation. Authors have ensured that the raw data will be retained in full for a reasonable period after publication and can be presented to editors and reviewers upon request. Where appropriate, datasets have been deposited in a publicly accessible repository Zenodo https://doi.org/10.5281/zenodo.17089263.

## Abbreviations

The following abbreviations are used in this manuscript:

OPCABG	Off-Pump Coronary Artery Bypass Grafting
Copeptin	C-terminal portion of provasopressin
BNP	B-type Natriuretic Peptide
NT-proBNP	N-terminal prohormone of BNP
ADH	Antidiuretic Hormone
AVP	Arginine Vasopressin
MAP	Mean Arterial Pressure
SBP	Systolic Blood Pressure
DBP	Diastolic Blood Pressure
DI	Diabetes Insipidus
